# LDL receptor related protein 1 is an adverse prognostic biomarker that correlates with stromal remodeling and macrophages infiltration in bladder cancer

**DOI:** 10.3389/fimmu.2023.1113756

**Published:** 2023-04-20

**Authors:** YiHeng Du, YiZheng Liu, Jin Cao, Xiang Jiang, Yi Wang, Jiang Yu, Bo Wang, XiZhi Wang, BoXin Xue

**Affiliations:** ^1^ Department of Urology, Suzhou Kowloon Hospital, Shanghai Jiao Tong University School of Medicine, Suzhou, China; ^2^ Department of Urology, The Second Affiliated Hospital of Soochow University, Suzhou, China; ^3^ Department of Pathology, Suzhou Kowloon Hospital, Shanghai Jiao Tong University School of Medicine, Suzhou, China

**Keywords:** bladder cancer, LRP1, cancer-associated fibroblasts, macrophages, Immune checkpoint blockage

## Abstract

**Introduction:**

Bladder cancer (BLCA) is a highly heterogeneous disease influenced by the tumor microenvironment, which may affect patients' response to immune checkpoint blockade therapy. Therefore, identifying molecular markers and therapeutic targets to improve treatment is essential. In this study, we aimed to investigate the prognostic significance of LRP1 in BLCA.

**Methods:**

We analyzed TCGA and IMvigor210 cohorts to investigate the relationship of LRP1 with BLCA prognosis. We utilized gene mutation analysis and enrichment to identify LRP1-associated mutated genes and biological processes. Deconvolution algorithms and single-cell analysis were used to understand the tumor-infiltrated cells and biological pathways associated with LRP1 expression. Immunohistochemistry was conducted to validate the bioinformatics analysis.

**Results:**

Our study revealed that LRP1 was an independent risk factor for overall survival in BLCA patients and was associated with clinicopathological features and FGFR3 mutation frequency. Enrichment analysis demonstrated that LRP1 was involved in extracellular matrix remodeling and tumor metabolic processes. Furthermore, the ssGSEA algorithm revealed that LRP1 was positively correlated with the activities of tumor-associated pathways. Our study also found that high LRP1 expression impaired patients' responsiveness to ICB therapy in BLCA, which was predicted by TIDE prediction and validated by IMvigor210 cohort. Immunohistochemistry confirmed the expression of LRP1 in Cancer-Associated Fibroblasts (CAFs) and macrophages in the tumor microenvironment of BLCA.

**Discussion:**

Our study suggests that LRP1 may be a potential prognostic biomarker and therapeutic target in BLCA. Further research on LRP1 may improve BLCA precision medicine and enhance the efficacy of immune checkpoint blockade therapy.

## Introduction

Bladder cancer (BLCA) is a common and serious disease worldwide, with approximately 80,000 new cases and 150,000 deaths each year (1). BLCA can be classified into two types: non-muscle invasive bladder cancer (NMIBC) and muscle-invasive bladder cancer (MIBC). Approximately 20% of all BLCA cases are diagnosed as MIBC, which has a higher risk of metastasis and a poor prognosis (2). NMIBC, if not detected and treated early, can invade the bladder wall and spread to neighboring organs or lymph nodes, leading to a worse prognosis and more aggressive treatment (3). The diversity in cellular components exhibited by MIBC leads to significant variability in cancer aggressiveness, progression, and response rates, making MIBC particularly difficult to treat. Thus, getting a better understanding about the heterogeneity of MIBC is of great importance for improving BLCA treatment.

The tumor microenvironment (TME), composed of both cellular and non-cellular components, plays a crucial role in tumor initiation and progression (4). Cancer-associated fibroblasts (CAFs), a type of stromal cell, have been shown to contribute to cancer growth and maintenance by secreting collagen, matrix metalloproteinases, and chemokines, and by remodeling the extracellular matrix (5). CAFs can also promote immunosuppression and cancer progression by interacting with macrophages in the TME, leading to the heterogeneity of TME and resistance to chemotherapy and immunotherapy. A better understanding of CAFs could help improve our understanding of the TME and identify potential therapeutic targets for BLCA.

Low-density lipoprotein receptor-related protein 1 (LRP1) is a type I transmembrane protein belonging to the low-density lipoprotein receptor family (6). It is mainly expressed in stromal and immune cells, including fibroblasts, monocytes, and macrophages (7). LRP1 plays important roles in regulating lipid homeostasis (8), glucose metabolism (9), and inflammation (10), and has been shown to be involved in extracellular matrix remodeling (11) and cancer progression (12). In this study, we conducted a comprehensive analysis of the role of LRP1 in BLCA using transcriptome profiling and single-cell RNA sequencing. Our results revealed the fundamental association of LRP1 with TME heterogeneity and the prognosis of BLCA patients. Further research on LRP1 could provide valuable insights for developing new therapeutic strategies for BLCA.

## Materials and methods

### Raw data acquisition

Gene expression quantification data were collected from the TCGA database for 408 BLCA patients. The method and use of this data comply with the appropriate guidelines and policies and can be accessed through the following website: https://portal.gdc.cancer.gov/. To validate the correlation between LRP1 and the activities of tumor-related pathways, additional data was obtained from the Gene expression Omnibus (GEO) datasets GSE13507 and GSE32894, which can be found at https://www.ncbi.nlm.nih.gov/geo/. Before analyzing the data, the gene expressions from the TCGA and GEO cohorts were transformed using Log2(expression+1) and were then normalized using the ‘Combat’ algorithm from the “SVA” package. Lastly, the IMvigor210 cohort was obtained from the R package ‘IMvigor210CoreBiologies’ (13) and was used for external validation in this study.

### Survival analysis

The difference in survival between patients was analyzed using the Kaplan-Meier (KM) method. The log-rank test and univariate Cox regression were used to calculate the P-value and hazard ratio (HR) with a 95% confidence interval (CI). Patients with missing or inappropriate survival information were excluded from the analysis. The KM curves were generated using the R language version 4.0.3 with the packages “ggrisk,” “survival,” and “survminer.”

### Independent risk analysis and nomogram construction

To identify independent risk factors for BLCA prognosis, univariate and multivariate Cox regression analysis was performed. The hazard ratio (HR) and 95% confidence interval (CI) for each variable were calculated using the R package “forest plot.” The results of the multivariate analysis were used to create a nomogram for predicting 1, 3, and 5-year overall survival (OS) using the “rms” R package. The nomogram is a visual representation that shows the mortality risk for a single patient based on the points assigned to each risk factor. The predictive accuracy of the nomogram was evaluated using the C-index and a calibration plot. The nomogram was created using the online bioinformatics tool “Assistant for Clinical Bioinformatics (aclbi tool)” (www.aclbi.com).

### Gene mutation analysis

To analyze the relationship between gene mutations and gene expression, the TCGA database was used to download somatic mutation data, which was then visualized using the “maftools” package of the R language. A waterfall plot was created to display mutation information for each gene, with annotations added to the top right of the plot using different colors to indicate different mutation types. The construction of the waterfall plot was accomplished using the aclbi tool. Finally, the TIMER 2.0 database (http://timer.cistrome.org/) (14) was used to analyze the relationship between gene mutations and gene expression.

### Acquisition and functional enrichment analysis of differentially expressed genes (DEGs)

The Limma package of R software analyzed the differentially expressed genes (DEGs) between the LRP1^high^ and LRP1^Low^ groups. The adjusted P-value was used to correct for false-positive results. DEGs were defined as genes with “Adjusted P < 0.05 and |Log2 (Fold Change)| >1”. The Gene Ontology was used to annotate gene functions, covering molecular function, biological pathways, and cellular components. The KEGG Enrichment Analysis was conducted to uncover high-level information about gene function. The R package ‘ClusterProfiler’ was used to analyze the GO functions and enrich KEGG pathways of potential targets. The aclbi tool was used to conduct the enrichment plot.

### Estimation of the TME

The immune cell infiltration was estimated by the ssGSEA algorithm. Information of immune inhibitors and immune stimulators were collected from the TISIDB database (15). Four algorithms, TIDE (16), MCP-COUNTER (17), xCELL (18), and EPIC (19), were used to estimate the abundance of CAFs in each sample from the TCGA BLCA cohort. The correlation between LRP1 expression and CAFs abundance was then obtained from the TIMER 2.0 database. The results from the TIDE database were used to predict the responsiveness of patients with different LRP1 expressions, and this was further verified using the IMvigor210 immunotherapy cohort.

### Single-cell RNA sequencing datasets acquisition and analysis

We obtained the raw data of single-cell transcriptome profiling (GSE190888) from the GEO database. The Seurat package conducted standard data preprocessing, where we calculated the percentage of the gene numbers, cell counts, and mitochondria sequencing count. We filtered out cells with fewer than 200 and more than 7000 detected genes and those with a high mitochondrial content (>20%). To normalize the library size effect in each cell, we scaled UMI counts using scale. factor = 10,000. Following the log transformation of the data, other factors, including “percent. mt”, “nCount_RNA,” and “nFeature_RNA,” were corrected for variation regression using the ScaleData function in Seurat (v3.0.2). The harmony algorithm was applied to remove the batch effects between different samples. After clustering cells with t-SNE, we identified signature genes in different cell populations and used the signature genes of fibroblasts and macrophages for deconvolution analysis with the ssGSEA algorithm. The single-cell RNA sequencing dataset (GSE130001 and GSE145281) was obtained from the TISCH (http://tisch.comp-genomics.org/home/) (20), a database providing detailed cell-type annotation at the single-cell level for the exploration of TME across different cancer types.

### Immunohistochemical (IHC) analysis

We identified stromal expression of LRP1 (Abways CY5215) in 40 tumor sections using the BenchMark GX automatic multifunctional immunohistochemistry staining system (Roche, Switzerland) with the OptiView DAB Detection Kit (Ventana, USA), following the manufacturer’s instructions. A secondary antibody labeled with horseradish peroxidase visualized the primary antibodies. Post-counterstaining was performed using Bluing Reagent after Hematoxylin counterstaining. To clarify LRP1 expression in fibroblasts, we labeled CD45(Abways, CY5311) negative S100A4 (Abways, CY5799) positive cells as fibroblasts, and used CD68 (Abways, AB3506) to identify immune infiltration of macrophages. Additionally, two pathologists evaluated the immunohistochemical results without knowledge of the patient’s information (Jiang Xiang & Cao Jin). The IHC score was calculated based on the staining intensity and the proportion of positive stromal cells, with the following criteria: [IHC score 1], weak staining in <50% or moderate staining in <20% of stromal cells; [IHC score 2], weak staining in ≥50%, moderate staining in 20-50%, or intense staining in <20%; [IHC score 3], moderate staining in ≥50% or intense staining in ≥20%. Cases with scores 2 or 3 were considered positive for each protein expression (21). We further calculated the percentage of LRP1-positive stromal area in cases that tested positive for LRP1 and divided these cases into high and low LRP1 expression groups using a reference point of 50%. Patient information is presented in **Table 1**.

**Table 1:** Patient information of the validation cohort.

LRP1	Gender		Age		Grade		T-stage		
	male	female	<65	≥65	High	Low	Ta-T1	T2	T3-T4
Low	11	4	6	9	5	10	11	3	1
High	18	7	4	21	24	1	4	15	6

### Statistical analysis

The statistical methods used in this study provide a comprehensive approach to analyze the data and draw conclusions about the relationship between LRP1 expression and BLCA prognosis. The KM survival analysis, Log-Rank test, and Cox regression analysis were used to evaluate the survival differences and identify independent risk factors for BLCA prognosis. The Wilcoxon test, Student’s t-test, and Kruskal-Wallis’s test were used to compare the differences between variables in two or more groups. The Fisher’s exact test was used to analyze the association between LRP1 expression and clinical features. All statistical analyses were conducted using the R language version 4.2.1, and a two-sided P-value less than 0.05 was considered significant.

## Results

### LRP1 expression is correlated with patients’ overall survival in TCGA and IMvigor210 cohorts

**Figure 1:**
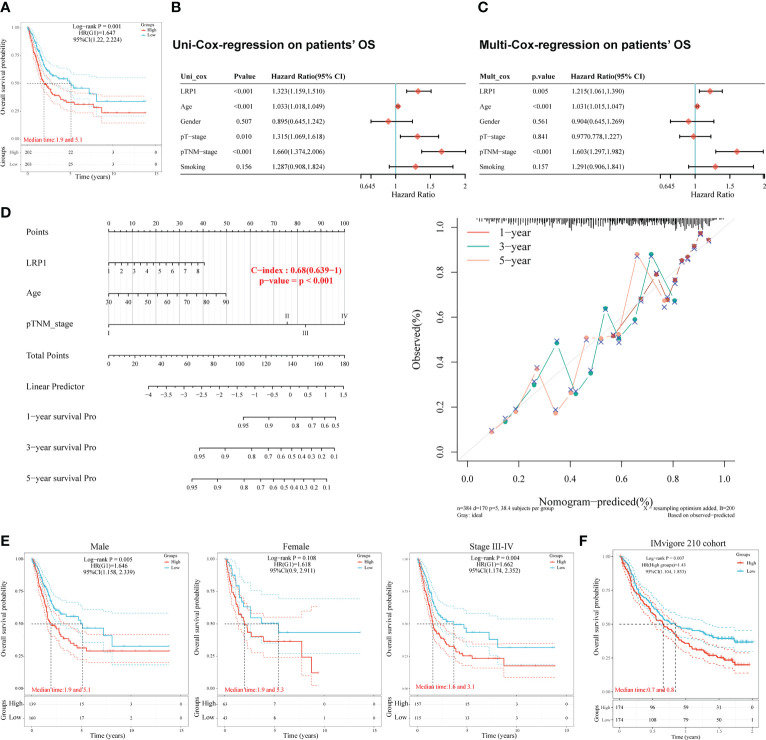
LRP1 is an adverse prognostic factor of BLCA patients. **(A)**. K-M survival analysis confirmed LRP1 as an adverse prognostic factor of BLCA patients’ OS (p=0.001). **(B, C)**. Univariate (p<0.001) and multivariate (p=0.005) Cox regression analysis suggested LRP1 as an independent risk factor to BLCA patients’ OS. **(D)**. The nomogram combining LRP1 expression level, age, and clinical stage predicted patients’ survival probability with a c-index of 0.680(0.639-1). **(E)**. Subgroup survival analysis emphasizes the crucial roles of LRP1 expression in male patients (p=0.005) and MIBCs with stage III-IV (p=0.004). **(F)**. The external validation cohort (IMvigor210) confirmed the detrimental role of LRP1 on BLCA patients’ OS (p=0.007).

To understand the impact of LRP1 on the prognosis of BLCA patients, we studied the relationship between LRP1 expression and patients’ OS in the TCGA BLCA cohort. Our findings showed that higher levels of LRP1 expression had a significant negative impact on patients’ OS (p=0.001), with patients expressing higher levels of LRP1 having lower median survival interval (**Figure 1A**). Further analysis using univariate (**Figure 1B**) and multivariate Cox regression (**Figure 1C**) revealed that LRP1 expression (p=0.005), age (p<0.001), and clinical stage (p<0.001) were independent predictors of patients’ OS. Based on the results of the multivariate Cox regression, we developed a predictive nomogram that visualizes patients’ survival probability based on different levels of LRP1 expression, age, and clinical stage. The calibration curve of the nomogram showed that it could accurately predict patients’ OS, with a c-index of 0.680 (0.639-1) (**Figure 1D**). Subgroup survival analysis showed that LRP1 negatively impacted the OS in male subgroup (p=0.005), with its detrimental effect being more pronounced in patients with advanced BLCA (Stage III-IV, p=0.004). These results suggested that LRP1 played a crucial role in male and MIBC patients (**Figure 1E**). Finally, our findings were validated by the IMvigor210 cohort, which showed that LRP1 had an adverse impact on the OS of BLCA patients (**Figure 1F**, p=0.007). Overall, these results confirm that LRP1 expression levels significantly reduce the OS of BLCA patients, especially in male and MIBC patients.

### LRP1 expression is closely associated with the clinicopathological characteristics of BLCA patients

**Figure 2:**
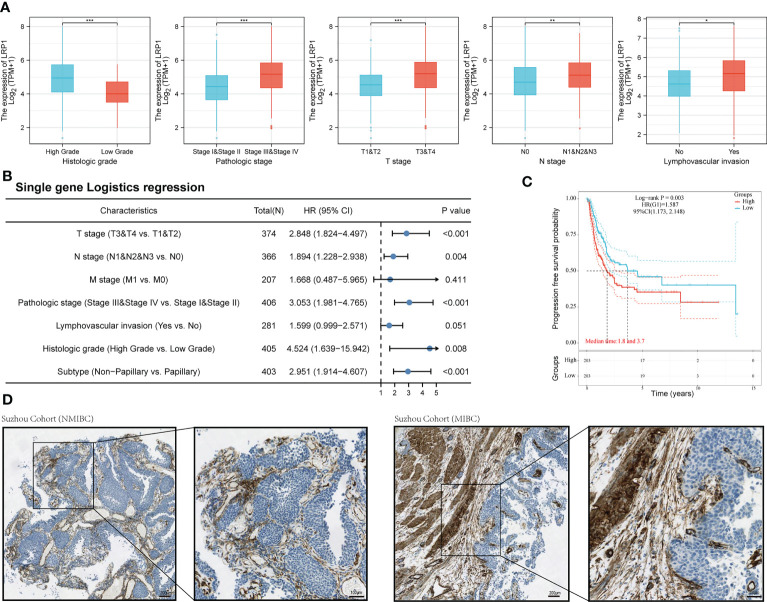
LRP1 expression is correlated with the pathological features of BLCA patients. **(A)**. LRP1 expression significantly correlated with the clinical pathological features of BLCA patients in the TCGA cohort. **(B)**. Single gene logistics analysis suggested increased LRP1 expression levels had advanced T-stage (p<0.001), increased risk of lymph node metastasis (p=0.004), and a greater tendency to present with a high-grade (p=0.008), non-papillary phenotype (p<0.001). **(C)**. LRP1 expression decreased the progression-free survival in TCGA BLCA patients (p=0.003). **(D)**. Immunohistochemistry analysis confirmed higher stromal expression proportion of LRP1 in MIBC compared with NMIBC, supporting that LRP1expressed at higher levels in non-papillary MIBC than in papillary tumors NMIBC. ***p<0.001, **p<0.01, *p<0.05.

We then looked at how LRP1 was related to the clinicopathological characteristics of BLCA patients. Our comparison showed that patients with higher LRP1 expression levels had higher tumor grade (p<0.001), advanced clinical stage (p<0.001), and were more likely to have lymph node metastasis (p<0.01) and lymphovascular invasion (p<0.05) **Figure 2A**. Logistic regression analysis confirmed that high LRP1 expression was more commonly found in high-grade non-papillary BLCA, and high LRP1 expression levels increased the risk of advanced T stage (p<0.001) and lymph node metastasis (p=0.004) (**Figure 2B**). The KM survival analysis showed that elevated LRP1 expression was linked to tumor progression and negatively affected patients’ progression-free survival (PFS) (p=0.003) (**Figure 2C**). To better understand the pattern of LRP1 expression and its correlation with pathological features, we used IHC analysis to examine LRP1 expression in clinical samples. The IHC results showed high proportions of LRP1 expression in the stromal components of BLCA and confirmed that LRP1 was broadly expressed in the stromal components in MIBC, further emphasizing the crucial role of LRP1 in MIBC (**Figure 2D**).

### LRP1 expression levels negatively correlate with the mutation frequency of FGFR and are involved in extracellular matrix remodeling and metabolism-related processes

**Figure 3:**
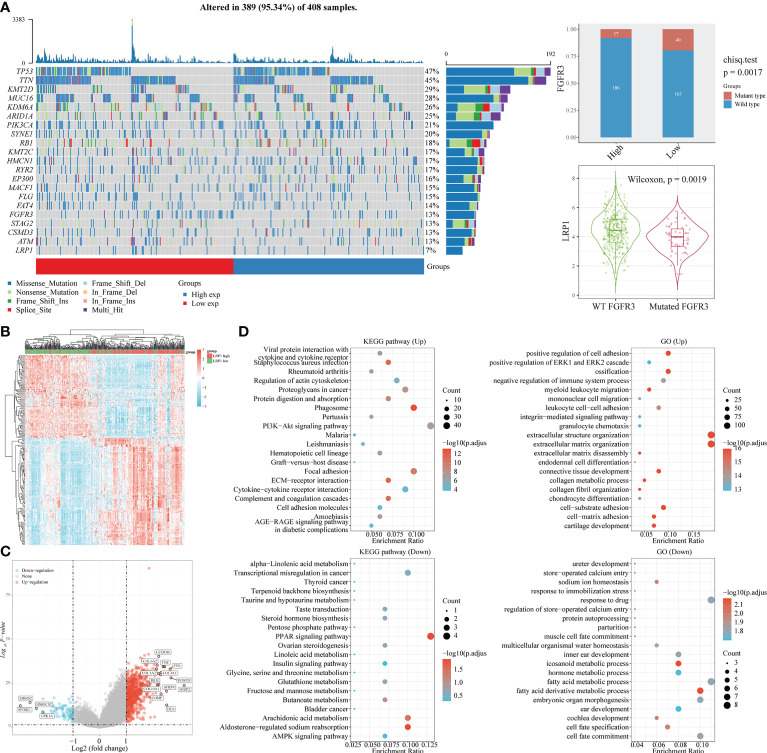
LRP1 expression is associated with FGFR3 mutation and extracellular matrix remodeling. **(A)** The top 20 most mutated genes in BLCA were listed in the waterfall plot grouped by LRP1 high and low expression, showing a significant difference of FGFR3 mutation frequency between the LRP1 high and low expression groups (p=0.0019). **(B)** DEGs between high and low LRP1 expression groups were displayed in the heatmap. **(C)** DEGs with highest fold changes, including SFRP2, POSTN,COMP, TNC and FN1, were shown in the volcano plot. **(D)** Functional enrichment analysis emphasized the significant roles of LRP1 in modulating the stromal microenvironment and metabolic processes.

With the expression of LRP1 found in the stromal cells of BLCA, we delved further into its relationship with stromal-related activities. Our mutation data analysis showed a significant negative correlation between LRP1 expression levels and the frequency of FGFR3 mutations in BLCA tissue. This result suggested that LRP1 expression levels were linked to the content of stromal component at the gene mutation frequency level since tumors with high frequency of FGFR3 mutations have been found to own little fibroblastic component and were usually papillary in differentiation (**Figure 3A**). To better understand the association between LRP1 and stromal component-related activities, we screened LRP1-related DEGs and conducted GO and KEGG enrichment analyses. The DEGs were shown in the heatmap and volcano plot (**Figures 3B, C**). GO and KEGG analyses showed that LRP1-related genes were significantly enriched in biological processes linked to extracellular matrix (ECM) remodeling, including ECM-receptor interaction, cell adhesion molecules, extracellular matrix organization, and cell-substrate adhesion. These processes play a crucial role in regulating inter-cellular adhesion and migration and are directly related to the migration and invasive ability of tumor cells. Additionally, we found that LRP1-related genes are also closely linked to metabolic activities such as hormones and fatty acids (**Figure 3D**). These results further highlighted that LRP1 expression in BLCA stroma was associated with biological processes that promote tumor progression, including extracellular matrix organization and tumor metabolism.

### An integrated BLCA cohort further confirmed that LRP1 expression is correlated with the diversity of the tumor microenvironment

**Figure 4:**
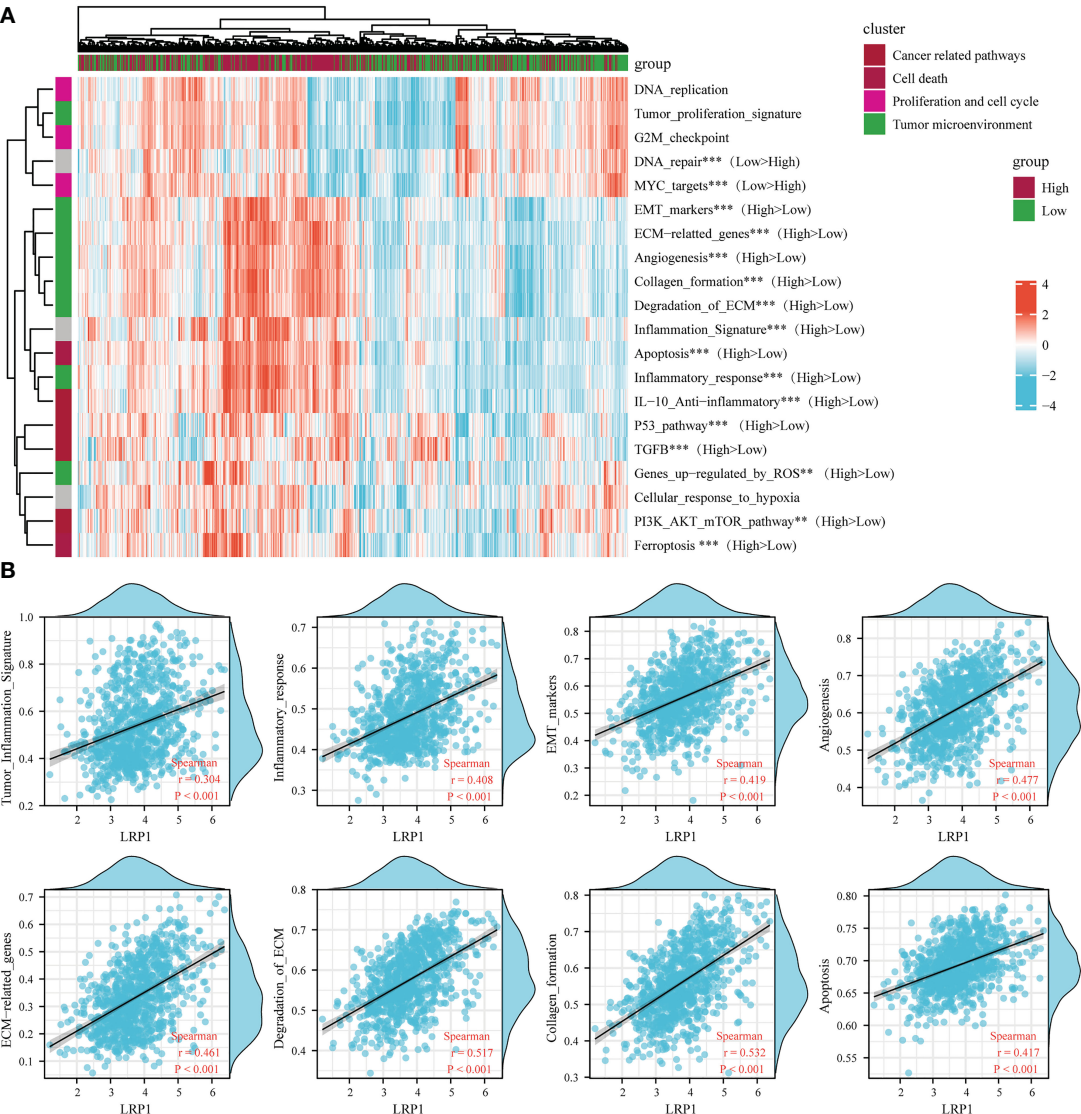
LRP1 expression in BLCA is related to multiple tumor-associated pathways. **(A)**. The ssGSEA algorithm confirmed the close relationship between LRP1 expression and multiple tumor-related signatures, including EMT, ECM remodeling, inflammation, TGF-beta activity, and DNA repair. **(B)**. The correlation test further displayed the crucial correlations of LRP1 expression with tumor inflammation signature (R=0.304 p<0.001), inflammatory response (R=0.408 p<0.001), EMT (R=0.419 p<0.001), angiogenesis(R=0.477 p<0.001), ECM related genes (R=0.461 p<0.001), ECM degradation (R=0.517 p<0.001), collagen formation (R=0.532 p<0.001), and apoptosis(R=0.417 p<0.001). ***p<0.001, **p<0.01.

To reinforce our findings from the TCGA database, we conducted a thorough analysis of the connection between LRP1 expression levels and pathways related to the tumor microenvironment (22) by merging two BLCA sequencing databases from GEO (GSE13507 and GSE32894). Our analysis revealed that the epithelial-mesenchymal transition (EMT) (p<0.001), degradation of the extracellular matrix (ECM) (p<0.001), collagen formation(p<0.001), angiogenesis (p<0.001), inflammatory signature (p<0.001), and activity in the PI3K-AKT-mTOR pathway(p<0.01) were stronger in tumors with high LRP1 expression, while DNA damage repair (p<0.001) and activity of MYC targets (p<0.001) were reduced (**Figure 4A**). The Spearman correlation analysis showed a significant positive correlation between LRP1 expression levels and tumor inflammation (R=0.304, p<0.001), inflammatory response (R=0.408, p<0.001), EMT (R=0.419, p<0.001), angiogenesis (R=0.477, p<0.001), ECM-related genes (R=0.461, p<0.001), ECM degradation (R=0.517, p<0.001), collagen formation (R=0.532, p<0.001), and apoptotic activity (R=0.417, p<0.001) (**Figure 4B**). The integrated cohort confirmed our findings, suggesting that LRP1 expression level can contribute to the diversity of the tumor microenvironment by influencing multiple pathways related to the tumor microenvironment and creating an environment conducive to tumor progression.

### LRP1 predicts immunotherapy responsiveness in BLCA associated with tumor-associated macrophage and CAFs infiltration

**Figure 5:**
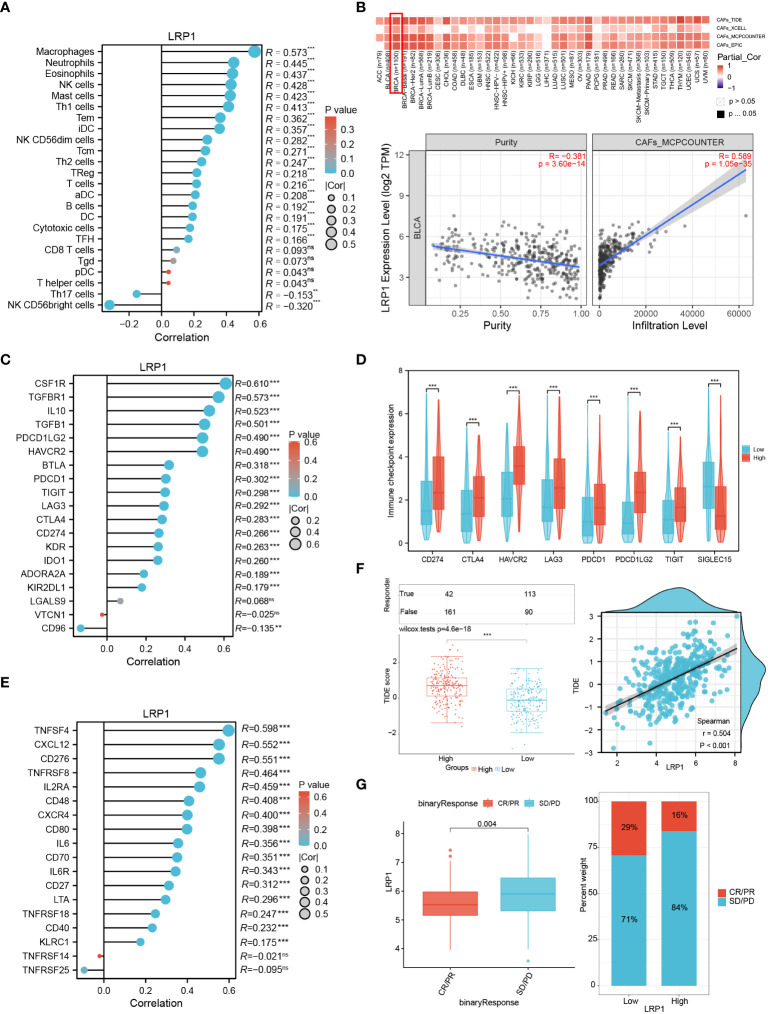
LRP1 expression affects immune infiltration and immunotherapy responsiveness. **(A)** ssGSEA algorithm clarified the significant correlation of LRP1 expression with immune infiltration, especially with the infiltration of macrophages (R=0.573,p<0.001) in BLCA. **(B)** TIDE, Xcell, TIMER and MCP-COUNTER algorithms confirmed the close relationship between LRP1 expression and CAFs abundance (MCP-COUNTER, R=0.589, P<0.001). **(C, D)** LRP1 was closely related to immune inhibitors, including CSF1R (R=0.610,p<0.001), IL10 (R=0.523,p<0.001), and TGFBR1(R=0.573,p<0.001), and immune checkpoint related genes, including CD274 (R=0.266, p<0.001), CTLA4 (R=0.283,p<0.001), PDCD1 (R=0.302,p<0.001) and TIGIT (R=0.298,p<0.001). **(E)** immune stimulators, containing TNFSF4 (R=0.598, p<0.001), CXCL12 (R=0.552, p<0.001), and CD276 (R=0.551, p<0.001) were closely related to LRP1 expression in BLCA. **(F)** TIDE prediction suggested significantly impaired responsiveness of LRP1 high expression BLCA patients to immune checkpoint inhibitors (p<0.001). **(G)** IMvigor210 immunotherapy cohort confirmed the adverse effect of LRP1 expression on BLCA patients' responsiveness to ICB therapy (p=0.004), with a lower proportion of CR/PR patients in the LRP1 high expression group. ***p<0.001,**p<0.01, ns not significant.

To gain a better understanding of how LRP1 impacts extracellular matrix and tumor microenvironment-related pathways, we investigated the levels of LRP1-related immune and stromal cell infiltration. By employing the ssGSEA algorithm, we observed a robust positive correlation between LRP1 and macrophage infiltration (R=0.573, p<0.001) in the TCGA BLCA cohort (**Figure 5A**). Moreover, using the TIDE, XCELL, MCP-COUNTER, and EPIC algorithms, we found that LRP1 was strongly associated with CAFs infiltration in BLCA (**Figure 5B**). Furthermore, we assessed the connection between immunity-related molecules and LRP1 expression levels in the TCGA BLCA cohort. Our analysis revealed that immunosuppressive molecules, including CSF1R, TGFBR1, and IL10 (**Figure 5C**), and immune checkpoint-related genes such as CD274, CTLA4, HAVCR2, LAG3, PDCD1, PDCD1LG2, and TIGIT were positively correlated with LRP1 expression. Conversely, LRP1 exhibited a significant negative correlation with SIGLEC15 (**Figure 5D**). Additionally, our investigation demonstrated that LRP1 was also highly associated with the expression of immunostimulators, such as TNFSF4 and CXCL12 in BLCA (**Figure 5E**). Overall, these findings underscore the critical role of LRP1 in regulating the immune microenvironment in BLCA. Given the close relationship between LRP1 and tumor immune modulators, we wanted to know if the expression level of LRP1 affects the responsiveness to immunotherapy in BLCA patients. To answer this question, we used the TIDE algorithm and found that BLCA patients with higher LRP1 expression had higher TIDE scores and tended to have weaker responsiveness to immunotherapy treatment (p<0.001) (**Figure 5F**). Our results from the IMvigor210 cohort also showed that LRP1 expression levels were significantly higher in patients who experienced progression or stable disease (PD/SD) compared to those who had complete or partial response (CR/PR) (p=0.004), and a smaller proportion of CR/PR patients had high LRP1 expression (**Figure 5G**). These results indicate that the expression level of LRP1 is critical in regulating the immune microenvironment of BLCA and negatively affects the responsiveness to immunotherapy in BLCA patients.

### Single-cell analysis showed that LRP1 was mainly expressed by CAFs and macrophages in the BLCA tumor microenvironment

**Figure 6:**
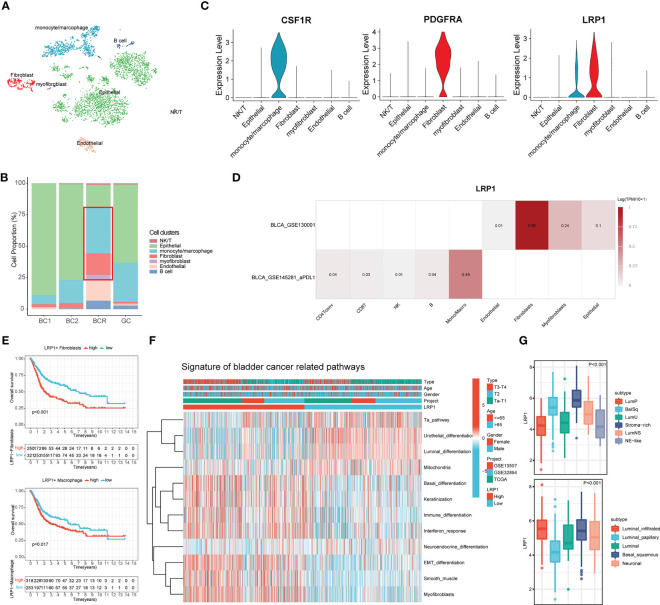
LRP1 expression is closely associated with fibroblasts and macrophages and predicts the molecular subtype of BLCA. **(A)** t-SNE plot distinguished different cell clusters in BLCA and GC. **(B)** Increased proportion of CAFs and macrophages were observed in recurrent BCs compared with primary BCs. **(C)** The violin plots revealed the expression of LRP1 by macrophages and CAFs. **(D)** Single-cell RNA sequencing from the TISCH database further confirmed the expression of LRP1 by fibroblasts myofibroblasts, and macrophages. **(E)** Deconvolution analysis based on cluster feature genes of single-cell RNA sequencing suggested a significant adverse impact of LRP1-expressing CAFs(p<0.001) and macrophages (p=0.017) on BLCA patients' OS. **(F, G)** Bladder cancer-related pathway and TCGA molecular subtype analysis further confirmed the higher LRP1 expression by stromal rich and basal squamous BLCAs.

To better understand the relationship between LRP1 and different cells, we looked more closely at LRP1-related cells at the single-cell level. We analyzed a single-cell dataset that included two cases of primary bladder cancer, one case of recurrent bladder cancer, and one case of glandular cystitis. We grouped the cells using the t-SNE algorithm (**Figure 6A**) and compared the proportion of different cell types in the tissues of the four cases (**Figure 6B**). The stacked barplot showed that the amount of macrophages, fibroblasts, and myofibroblasts was significantly higher in the recurrent BLCA tissues, which suggests that these cells are closely related to the recurrence and progression of BLCA. When we looked at the expression of LRP1 in different cell types, we found that LRP1 was mainly expressed in fibroblasts and macrophages (**Figure 6C**). We confirmed these results with single-cell sequencing data from two BLCA datasets in TISCH, which showed that LRP1 was significantly more highly expressed in macrophages and fibroblasts in the TME of BLCA (**Figure 6D**). These results were consistent with the link between LRP1 and immune cell infiltration that we found in our previous analysis. Next, we evaluated the impact of LRP1-positive fibroblasts and macrophages on the outcome of BLCA patients using the ssGSEA deconvolution algorithm. The results suggested that both LRP1-positive fibroblasts (p<0.001) and macrophages (p=0.017) have a significant negative impact on the OS of BLCA patients (**Figure 6E**). By comparing BLCA-related signatures, we found that bladder tumors with high LRP1 expression tend to have basal differentiation, a greater amount of fibroblastic and smooth muscle cells, and a larger immune infiltration (23). In contrast, BLCAs with low LRP1 expression tend to be more papillary and have luminal differentiation, with higher levels of mitochondrial activity. We also discovered that BLCAs have different differentiation, stromal, and immune cell infiltration levels depending on the level of LRP1 expression, which suggested that LRP1 expression levels may be a predictor of the molecular subtype of BLCA (**Figure 6F**). Finally, we confirmed the expression of LRP1 in BLCA with different molecular subtypes in the TCGA cohort. The results supported the association between LRP1 and basal differentiation and stromal infiltration, showing that LRP1 was expressed at relatively higher levels in BLCAs with basal and stromal-rich subtypes (**Figure 6G**). These results confirm that the harmful effect of LRP1 on the outcome of BLCA is associated with the behavior of CAFs and macrophages in the BLCA microenvironment, and that LRP1 expression is linked to the molecular subtype of BLCA.

### The expression of LRP1 by CAFs and its close relationship with macrophage infiltration was validated in a real-world validation cohort

**Figure 7:**
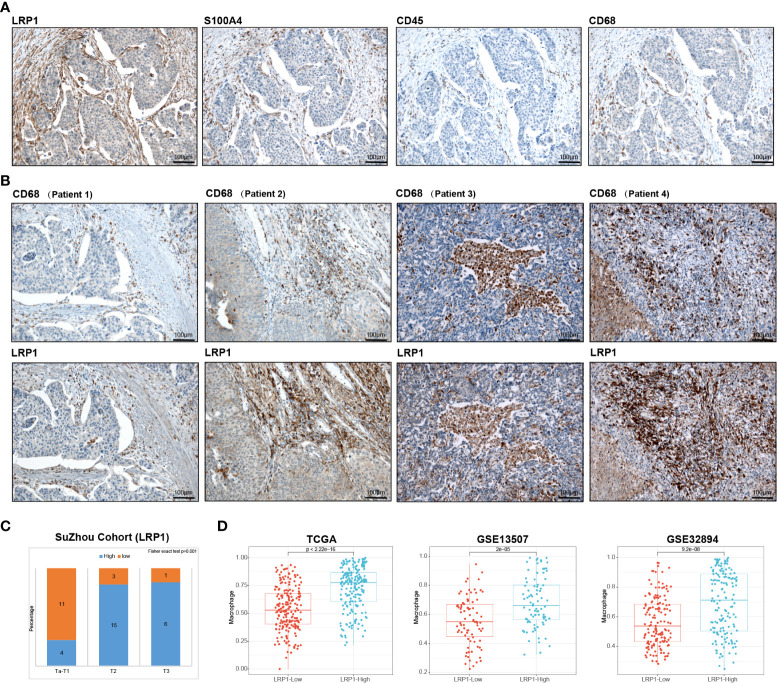
Validation of bioinformatics results by immunohistochemistry. **(A)** Immunohistochemistry analysis in the Suzhou cohort verified that LRP1 was expression in S100A4 positive and CD45 negative fibroblasts. **(B)**. IHC results from 4 individual BLCA patients further confirmed the expression of LRP1 by CAFs and macrophages **(C)**. Stromal LRP1 expression proportion was significantly correlated with the tumor stage in the Suzhou cohort. **(D)**. The immune deconvolution analysis further verified the correlation between LRP1 expression and macrophage infiltration in TCGA, GSE13507, and GSE32894 cohorts.

We conducted IHC tests on 40 postoperative BLCA sections of varying clinical stages from Suzhou Kowloon Hospital. Our results showed that LRP1 was primarily expressed in areas that tested positive for S100A4 and negative for CD45, indicating its expression in fibroblasts in the desmoplastic areas of BLCA (**Figure 7A**). Subsequently, we displayed typical IHC results from 4 BLCA patients, whose tumors were either rich in fibroblasts or highly infiltrated by CD68+ macrophages. The results from patient 1 and 2 demonstrated the idea that LRP1 was expressed by CAFs. While the results from patient 3 and 4 supported that macrophage also expressed LRP1, with the expression of LRP1 and CD68 highly overlapped in infiltrated immune cells, especially in patient 3 (**Figure 7B**). Additionally, we confirmed a significant correlation between stromal LRP1 expression proportions and clinical pathological features of patients (**Figure 7C**). By applying two datasets from the GEO (GSE13507 and GSE32894), we further verified the bioinformatic analysis in TCGA, further demonstrating the correlation between LRP1 expression and macrophage infiltration (**Figure 7D**). These results supported the expression of LRP1 in fibroblasts and macrophage, as revealed by our bioinformatics analysis.

## Discussion

BLCA is a common and serious form of urinary system cancer that is becoming more prevalent globally. BLCA can be divided into two main categories based on the depth of tumor cell invasion: NMIBC and MIBC. Although MIBC makes up only 20% of BLCA cases, it is responsible for the majority of deaths due to its poor prognosis and tendency to spread. MIBC is a highly heterogeneous form of cancer, with genomic, transcriptional, and cellular differences both within and between tumors. Despite the clonal initiation of BLCA, the progression and spread of BLCA can be influenced by changes in the surrounding tumor microenvironment, which can affect the behavior of tumor cells through the release of various chemicals, cytokines, and extracellular particles (24, 25). Recent advances in the treatment of BLCA have included the use of immune checkpoint inhibitors in the treatment for advanced BLCA (26), highlighting the importance of the tumor microenvironment in the development and outcome of BLCA. Further research into the tumor microenvironment has the potential to lead to new treatment options for BLCA.

LRP1 is a protein involved in endocytosis and the regulation of multiple signaling pathways. LRP1 binds to various structurally and functionally diverse ligands, and the endocytosis of these ligands can activate a range of pathways, such as PDGF, TGFβ1, Wnt, APOE, BMPs, and PPAR-γ signaling (27). Its broad expression and multifunctionality make LRP1 involved in many physiological and pathological processes, including the regulation of the extracellular matrix, transportation across the blood-brain barrier, coagulation, inflammation, lipid metabolism, Alzheimer’s disease, and atherosclerosis. Recent studies have shown the prognosis-related role of LRP1 in various types of cancer, including BLCA (28). Li X et al. identified LRP1 as a metabolism-related gene that is associated with the survival of BLCA (29), and Xing P et al. revealed that LRP1 was involved in regulating the immune infiltration level in BLCA (30). However, the expression pattern and the way how LRP1 expression influenced the prognosis of BLCA have not been well addressed. In the present study, we explored the prognostic and microenvironment-regulating role of LRP1 in BLCA through a comprehensive bioinformatic analysis of its relationship with immune infiltration levels. Our findings confirmed the previous findings, suggesting that LRP1 has a detrimental effect on the prognosis of BLCA and plays a key role in regulating the extracellular environment and immune activities in BLCA. Through validation in a real-world cohort of BLCA patients, we further demonstrated that stromal LRP1 expression was positively correlated with the abundance of fibroblastic components in BLCA, highlighting the influence of LRP1 on BLCA prognosis may function by CAFs.

The expansion of stromal fibroblast numbers in the TME can result from proliferation and is known as “stromagenesis,” which generates pro-tumorigenic fibroblasts (31). Our previous study also demonstrated that the continuous increase of CAFs abundance had a significant impact on the advancement and prognosis of BLCA (32). LRP1, expressed by CAFs, was found in the present study to be significantly involved in pathways that resulted in extracellular matrix remodeling, including extracellular matrix organization and focal adhesion. The expression pattern of LRP1 in BLCA revealed by immunohistochemical assays further indicated that LRP1 expression covered the stromal fibroblastic area, and a high level of LRP1 expression accompanied an increased number of fibroblastic tissues in BLCA, indicating the involvement of LRP1 in stromagenesis which significantly added to the heterogeneity of the TME. Additionally, the ssGSEA algorithm revealed a significant negative effect of LRP1-expressing CAFs on patient survival, further indicating that LRP1 expression was correlated with the generation of pro-tumor fibroblasts. With the above evidence, LRP1 may become an attractive option for stroma-focused anti-cancer intervention targeting fibroblast expansion in BLCA.

Molecular targeted therapy is a promising approach to treating BLCA, with FGFR3 being one of the most commonly mutated genes and thus an ideal target. Studies have shown that FGFR3 mutations in BLCA are closely linked to clinical and pathological staging. Specifically, FGFR3 mutations are significantly associated with NMIBC, lower pT stage, Grade 1-2, absence of carcinoma *in situ* (CIS), pN0, and low levels of p53. Tumors with FGFR3 mutations typically display higher levels of FGFR3 expression, leading to the activation of the luminal-papillary pathway. Consequently, FGFR3 mutations are commonly found in the papillary and luminal subtypes of bladder tumors (33). Our research uncovered a significant negative correlation between the expression levels of LRP1 and the status of FGFR3 mutations in BLCA, indicating that tumors with FGFR3 mutations generally have lower LRP1 expression. With further results showing that reduced LRP1 expression was associated with increased activity in the Ta and luminal differentiation pathways, we believed that the possible mechanism of changing LRP1 expression by the mutation of FGFR3 was due to the over-expression of FGFR3 and the activation of Ta and luminal differentiation pathways in FGFR3 mutated BLCAs. Therefore, our study highlighted the importance of LRP1 expression levels in guiding FGFR3-targeted molecular therapy.

In addition to CAFs, macrophages also play a crucial role in regulating the tumor microenvironment. Tumor-associated macrophages (TAMs) are the main immune cells that infiltrate the tumor microenvironment. TAMs have been extensively studied for their pro-tumor activities, including promoting tumor initiation, angiogenesis, metastasis, drug resistance, and immune suppression against tumors. TAMs interact with CAFs to exert immunosuppressive and pro-tumorigenic functions, leading to an immune suppressive tumor microenvironment. In the present study, we identified the expression of LRP1 by CAFs and macrophages in BLCA by IHC analysis. With further exploration on the role of LRP1 by bioinformatic analysis, our study showed significant impact of LRP1 expression on the activities of angiogenesis, collagen formation and degradation, EMT and inflammatory signature, indicating that LRP1 expressing CAFs and macrophages played a critical role in regulating the stromal and immune remodeling in BLCA. LRP1 expression also showed strong effects on the expression of immune inhibitors, including CSF1R, IL10, and TGFBR1, and immune checkpoint related genes, including CTLA4, CD274, PDCD1 and LAG3. Along with the prediction by TIDE algorithm and the validation by IMvigor210 cohort, our findings indicated that BLCA with high LRP1 expression tended to have lower responsiveness to immune checkpoint blockade therapy, highlighting the involvement of LRP1 expressing CAFs and macrophages in affecting the responsiveness of BLCA patients to ICB therapy. A previous study showed that liposomal therapy targeting macrophage LRP1 effectively suppresses the crosstalk between tumor metabolism and immune evasion *via* glycolysis inhibition and immune normalization, resulting in reduced lactic acid production and switching TAMs to an anti-tumor phenotype (34). This reinforces the anti-tumor function of the effector CD8+ T cells. These results further emphasized the critical roles of LRP1 in regulating the immune microenvironment and highlighted its potential as a promising therapeutic target for enhancing the efficiency of ICB therapy.

In addition, we also analyzed the difference in LRP1 expression levels on patient prognosis in different subgroups of BLCA. From the subgroup survival analysis of the TCGA database, we found that patients with high and low expression of LRP1 possessed significantly different OS patients with T3 and T4 stages. Limited by the patient number of the validation cohort, we did not obtain significant survival differences in our validation cohort. However, it was evident by the immunohistochemical results that high stromal expression proportion of LRP1 was closely associated with the significant increase in the abundance of stromal components in MIBC, implying that LRP1 high expression has a fundamental relationship with the heterogeneity of BLCA. Moreover, we found a correlation between the expression level of LRP1 and the molecular subtype of BLCA through the analysis of BLCA-related signaling pathways and different BLCA molecular typing systems based on the TCGA cohort. Our data showed that BLCAs with high LRP1 expression often showed stromal-rich or basal-squamous subtype, which remarkably agrees with the ssGSEA results depicting BLCA-related signaling pathways. These results further emphasized the association between LRP1 expression levels and the stromal components within BLCA, indicating that LRP1 expression levels could potentially predict the molecular subtypes of BLCA.

With all the above results summarized by our manuscript, we have comprehensively explored the roles of LRP1 in the tumor microenvironment of BLCA through an integrated bioinformatics analysis and immunohistochemical validation and confirmed the expression of LRP1 in CAFs and macrophages. Along with the results of previous literature, we systematically summarized the potential functions played by LRP1 in the tumor microenvironment of BLCA. However, due to the long coding sequence of LRP1, it is difficult for us to investigate the specific functions of LRP1 in CAFs and macrophages by experimental approaches such as lentivirus packaging or siRNA interference. The specific functions of LRP1 in BLCA are to be verified by *in-vitro* and *in-vivo* experiments. Due to the presence of post-transcriptional modifications, the relationship between the gene expression level and protein level of LRP1 also needs to be verified by further experiments. Limited by the number of cases in our real-world validation cohort, the existing findings require further validation in larger cohorts. Finally, since the existing bioinformatics algorithms cannot be entirely accurate in assessing the content of the tumor microenvironment, more accurate algorithms need to be developed and validate the current results.

## Conclusion

This manuscript provides insight into the close association of LRP1 with stromal remodeling and immune infiltration in the tumor microenvironment of BLCA through systematic bioinformatics analysis, literature search, and real-world cohort validation. Our results suggested a significant adverse effect of high LRP1 expression in the tumor microenvironment on the prognosis of BLCA patients. By the crucial relationship of LRP1 expression with the infiltration of macrophages and CAFs abundance, our manuscript indicated that LRP1 was an immune suppressive factor in the tumor microenvironment of BLCA, further influencing patients’ responsiveness to ICBs therapy. We believe that LRP1 could be a potential target for BLCA treatment and promoting the responsiveness of BLCA immunotherapy after further study.

## Data availability statement

The original contributions presented in the study are included in the article/supplementary material. Further inquiries can be directed to the corresponding authors.

## Ethics statement

The studies involving human participants were reviewed and approved by Medical Ethics Committee of Suzhou Kowloon Hospital. The patients/participants provided their written informed consent to participate in this study.

## Author contributions

YD and YL had an equal contribution to this manuscript. YD and BX designed the study. YD and YL conducted the bioinformatics and statistical analysis. XJ and JC did the immunohistochemistry analysis and the corresponding IHC scoring. YW and JY made the manuscript and figure editing. BW and XW revised the manuscript. XW and BX provided the administrative approval. All authors contributed to the article and approved the submitted version.
